# Protocol of the Snuggle Bug/Acurrucadito Study: a longitudinal study investigating the influences of sleep-wake patterns and gut microbiome development in infancy on rapid weight gain, an early risk factor for obesity

**DOI:** 10.1186/s12887-021-02832-8

**Published:** 2021-08-31

**Authors:** Megan E. Petrov, Nana Jiao, Sarada S. Panchanathan, Elizabeth Reifsnider, Dean V. Coonrod, Li Liu, Rosa Krajmalnik-Brown, Haiwei Gu, Laurie A. Davidson, Robert S. Chapkin, Corrie M. Whisner

**Affiliations:** 1 Edson College of Nursing and Health Innovation, Arizona State University, 550 N. 3rd Street, Suite 301, Phoenix, AZ 85004 USA; 2 Valleywise Comprehensive Health Center – Phoenix (Pediatric Clinic), 2525 E. Roosevelt St., Phoenix, AZ 85008 USA; 3 College of Medicine Phoenix, University of Arizona, Phoenix, AZ 85007 USA; 4 Valleywise Health, Department of Obstetrics and Gynecology, 2525 E. Roosevelt St., Phoenix, AZ 85008 USA; 5 Biodesign Institute, Arizona State University, 1001 S. McAllister Ave BDA230B, Tempe, AZ 85287 USA; 6 Biodesign Swette Center for Environmental Biotechnology, Arizona State University, 1001 S. McAllister Ave, PO Box 875701, Tempe, AZ 85287 USA; 7 College of Health Solutions, Arizona State University, 550 N. 3rd. Street, Suite 501, Phoenix, AZ 85004 USA; 8 Department of Nutrition and Food Science, Program in Integrative Nutrition and Complex Diseases, Texas A&M University, 2253 TAMU, 112 Cater-Mattil, College Station, TX 77843 USA

**Keywords:** infant sleep, Actigraphy, circadian rhythm, sleep-wake pattern, Gastrointestinal microbiota, Rapid weight gain, child overweight; time varying effect models

## Abstract

**Background:**

Overweight, obesity, and associated comorbidities are a pressing global issue among children of all ages, particularly among low-income populations. Rapid weight gain (RWG) in the first 6 months of infancy contributes to childhood obesity. Suboptimal sleep-wake patterns and gut microbiota (GM) have also been associated with childhood obesity, but little is known about their influences on early infant RWG. Sleep may alter the GM and infant metabolism, and ultimately impact obesity; however, data on the interaction between sleep-wake patterns and GM development on infant growth are scarce. In this study, we aim to investigate associations of infant sleep-wake patterns and GM development with RWG at 6 months and weight gain at 12 months. We also aim to evaluate whether temporal interactions exist between infant sleep-wake patterns and GM, and if these relations influence RWG.

**Methods:**

The Snuggle Bug/ Acurrucadito study is an observational, longitudinal study investigating whether 24-h, actigraphy-assessed, sleep-wake patterns and GM development are associated with RWG among infants in their first year. Based on the Ecological Model of Growth, we propose a novel conceptual framework to incorporate sleep-wake patterns and the GM as metabolic contributors for RWG in the context of maternal-infant interactions, and familial and socio-physical environments. In total, 192 mother-infant pairs will be recruited, and sleep-wake patterns and GM development assessed at 3 and 8 weeks, and 3, 6, 9, and 12 months postpartum. Covariates including maternal and child characteristics, family and environmental factors, feeding practices and dietary intake of infants and mothers, and stool-derived metabolome and exfoliome data will be assessed. The study will apply machine learning techniques combined with logistic time-varying effect models to capture infant growth and aid in elucidating the dynamic associations between study variables and RWG.

**Discussion:**

Repeated, valid, and objective assessment at clinically and developmentally meaningful intervals will provide robust measures of longitudinal sleep, GM, and growth. Project findings will provide evidence for future interventions to prevent RWG in infancy and subsequent obesity. The work also may spur the development of evidence-based guidelines to address modifiable factors that influence sleep-wake and GM development and prevent childhood obesity.

## Background

### Early rapid weight gain in infancy contributes to childhood obesity

Early childhood obesity is a serious health problem. In 2019, 38.2 million U.S. children less than 5 years old were overweight or obese, with greater prevalence among low- and middle-income families [1]. Of those under 2 years, 8.1% had high weight-for-recumbent length (i.e., at or above the 95th percentile) [2]. Further, obese children are more likely to become obese adults [3]. Rapid weight gain (RWG) in the first 6 months of infancy, defined as a greater than 0.67 positive change in weight-for-age Z-score (difference between centile lines on standard growth charts) [4], is associated with greater total weight gain from 0 to 12 months and greater weight-for-length and weight-for-age percentiles at 36 months [5]. It also increases the odds of obesity across the life course [6–9] and relates to adverse health outcomes later in life, including hypertension, diabetes, and cardiovascular diseases [10]. Further, RWG in the first 3 months is more predictive of poor cardiometabolic outcomes than any other 3-month period in the first year of life [11]. Despite robust, globally diverse cohort data accounting for numerous confounding factors present from conception to birth on childhood obesity, few studies have focused on RWG determinants and timing in the birth-to-6-month period.

### Suboptimal sleep-wake patterns are important risk factors for childhood obesity but understudied in infants in relation to RWG

Systematic reviews and meta-analyses conclude that short sleep duration is associated with childhood overweight/obesity [12, 13]. Prospective cohort studies suggest that short sleep duration (parent reported < 12 h) in early infancy is associated with greater body mass index (BMI) at 24 months [13], a two-fold odds of childhood obesity [14], and greater weight-for-length and excess weight gain by 24 months [15]. However, evidence suggesting that longer sleep duration in infancy is associated with healthier body composition remains uncertain [16]. Sleep is a multidimensional construct composed of other components beyond sleep duration that contribute to obesity risk, including sleep-wake timing and patterns (e.g., circadian rhythmicity). Greater nap frequency in infancy [17] and more rapid sleep stage development (i.e., active and quiet) among preterm infants [18] also predict subsequent growth in length and favorable weight outcomes, respectively. Further, data from three randomized controlled trials suggest that infant sleep promotion (i.e., adequate sleep duration, not feeding to soothe baby to sleep) influences adiposity. Infants receiving interventions with a sleep component had lower BMI z-scores [19, 20] and weight-for-length percentiles [21], were less likely to be overweight/obese [19], and had slower weight gain rates [19] than non-sleep interventions (follow-up from 6 months to 5 years). However, no studies have explored associations between sleep-wake patterns and RWG, with the exception of our single, previous study [5]. Our results indicated that newborns (1 month) with later bedtimes (≥10:00 PM) were more likely to experience RWG in their first 6 months than newborns who went to bed earlier [5]. Further, newborns taking more daytime naps were significantly less likely to experience RWG [5]. Also, previous studies have rarely used actigraphic monitoring (i.e., movement-based recording with a wearable sensor) of infants to provide a comprehensive, prospective assessment of sleep patterns [22], particularly in association with early infant growth. One exception was a prospective study that found shorter nocturnal sleep duration measured via actigraphy at 8 months of age was associated with higher odds for greater weight-for-length at 24 months [15].

### Gut microbiota (GM) is associated with childhood obesity, but little is known about microbial influences on infant RWG

The majority of GM development including colonization and maturation occurs within the first 3 years of life [23]. Previous studies have linked breast vs. formula feeding [24–30], birth delivery mode [26, 30, 31], maternal pre-pregnancy weight [30, 32], and perinatal antibiotic exposure [33, 34] to unique differences in the ecology of the infant’s commensal GM. Two case-control studies matching participants for mode of birth delivery, birth weight, breastfeeding duration, antibiotic exposure, and gestational age suggest that decreased *Bifidobacteria* relative abundance, particularly at 3 months [35], is associated with childhood obesity [35, 36]. Increases in *S. aureus* relative abundance at 6 and 12 months have been associated with overweight at 7 years [35], while greater relative abundance of *B. fragilis* at 6 months was linked to overweight at 10 years [36]. Elevated counts of *B. fragilis* as early as 1 month have been associated with higher BMI z-scores across ages 1–10 years [37]. Differential trajectories of microbial communities are established during development [35] and up to 10% of GM fluctuate throughout the day [38]. Their influence on infant growth may vary depending on environmental exposures. Recent data have indicated that male and female infants differ in their response to GM changes with regard to early-life weight gain [39] and that maternal-child interactions play an important role in establishing GM communities and influencing infant growth [34, 40]. Delays in colonization of beneficial microbes in infancy are also associated with adiposity [41]. However, gut microbial influences on infant RWG are understudied. In addition, to date no large scale longitudinal studies have defined how early postnatal development of the gut microbiota is influenced by environmental factors and how, in turn, the microbiota influences host gene expression.

### Evaluation of the influence of the interaction between sleep-wake patterns and GM development on infant growth is scarce

Though sleep loss, fragmentation, and circadian misalignment are proposed stressors with the potential to influence GM and induce gut dysbiosis [42], limited pediatric studies on sleep-wake pattern and GM relationships exist. One study of older infants found that actigraphy-assessed nocturnal sleep duration at 6 months was associated with changes in GM beta-diversity [43]. Sleep metrics at 12 months were associated with GM beta-diversity. Specifically, greater fragmentation was associated with greater *Bacteroides* and lower *Lachnospiraceae* prevalence, and greater bedtime variability was associated with greater *B. longum* yet lower prevalence of other *Bifidobacterium* species [43]. Among adults, a within-subject crossover study (*n* = 9) found that 2 nights of partial sleep deprivation (4.25 vs. 8.5 h) increased the Firmicutes:Bacteroidetes ratio, led to greater relative abundance of *Coriobacteriaceae* and *Erysipelotrichaceae* families and lower relative abundance of *Tenericutes* [44]. Conversely, a study of 11 participants who underwent two rounds of partial sleep restriction (i.e., 4 nights with 4 h followed by 5 nights of 12 h) found no significant changes in GM composition [45]. A cross-sectional study of 37 community-based older adults found that better sleep quality was associated with greater *Verrucomicrobia* and *Lentisphaerae* relative abundance [46]. To our knowledge, there are, to date, no studies of the GM as a mediator for circadian sleep-wake disruption and subsequent infant RWG.

## Methods/design

### Theoretical framework

**Fig. 1:**
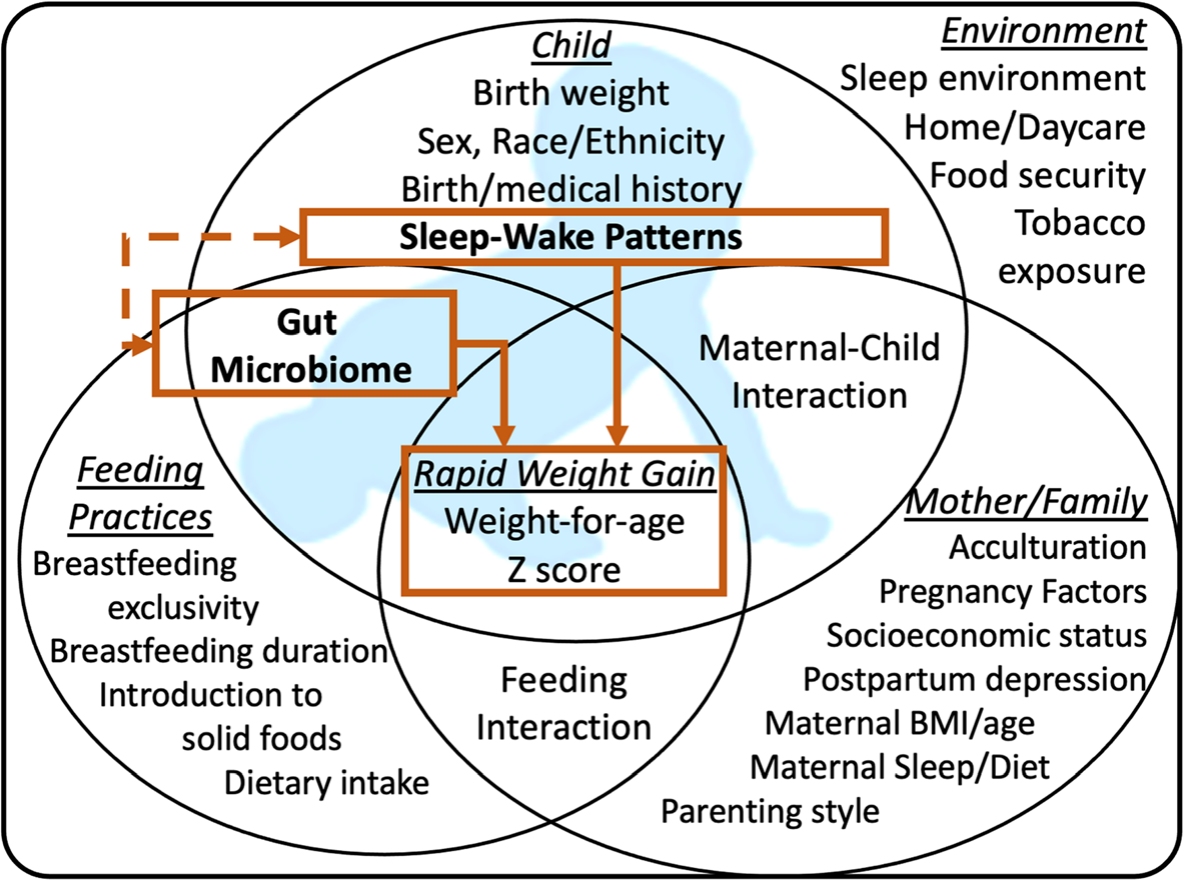
Ecological model of rapid weight gain

The Ecological Model of Growth (EMG) combines human ecology and epidemiology to evaluate factors that influence child health outcomes in the context of broader environmental constructs (parent, family, and home) [47, 48]. It has been validated as an effective tool for studying maternal and child factors that contribute to infant growth [49–51], but has not been used to study how EMG constructs influence RWG via the GM and sleep. Based on the EMG, we propose a conceptual framework that incorporates sleep-wake patterns and the GM as metabolic contributors for RWG in the context of socio-physical environments, to early-onset childhood obesity (Fig. 1). In the framework, major interactions to be explored or controlled for are (1) GM variation in response to child feeding mode and sleep-wake patterns, (2) maternal-child interactions (e.g., influence over sleep and soothing habits), (3) feeding interactions (e.g., parenting style, scheduled/on-demand feeding), and (4) environmental exposures.

### Aim and design

This study is designed as a five-year observational longitudinal study to investigate how 24-h sleep-wake patterns and GM influence RWG among infants in their first year. In this study, we aim to:

1. Investigate associations of infant sleep-wake patterns with subsequent RWG at 6 months and weight gain at 12 months using Time-Varying Effect Models (TVEM). We hypothesized that infants with suboptimal sleep-wake patterns (e.g., short sleep duration, poorer sleep quality [e.g., sleep percent, number of long awakenings], later bedtimes, and greater sleep-wake timing variability) will be more likely to experience RWG at 6 months and have greater total weight gain at 12 months than infants with optimal sleep-wake patterns.
2. Investigate associations of GM development with RWG at 6 months and weight gain at 12 months. We hypothesized that infants with lower GM diversity, decreased relative abundance of beneficial microbes (e.g., *Bifidobacteria*), and increased pathogen abundance (e.g., *Staphylococcus*) across time will be more likely to experience RWG at 6 months and have greater total weight gain at 12 months.
3. Evaluate whether temporal interactions exist between infant sleep-wake patterns and GM development, and if these relations influence RWG. We hypothesize that (1) infants with short sleep duration, poorer sleep quality, later bedtimes, and greater sleep-wake timing variability will exhibit lower GM diversity, decreased relative abundance of beneficial microbes, and increased pathogen abundance than infants with optimal sleep-wake patterns; and (2) infant sleep-wake patterns and GM development will have a synergistic effect on RWG, with suboptimal development of both factors increasing risk for RWG compared to either factor independently.

### Power analysis and target population

**Fig. 2:**
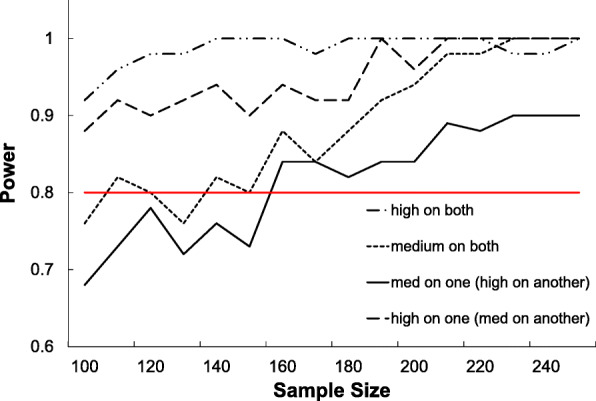
Power simulations for Aims 2 and 3. Each point is an average of 100 simulations

Using the Hedeker et al. approach [52], for a test of a between groups linear trend difference (comparing normal infant growth to those with RWG), equal sample sizes, 6 time points, a correlation among the repeated measures of 0.5, a compound symmetry structure, a medium effect size of one third of a standard deviation, an α of 0.05, and a power of 0.8, a total sample size of 184 participants (includes for estimated 20% attrition) is needed for Aim 1. To power for Aims 2–3, we reduced all EMG constructs (Fig. 1) into a single virtual covariate to facilitate computation. We then generated simulated data based on a mixed effect logistic regression model that included the virtual covariate, a sleep pattern variable and a GM diversity variable, each randomly drawn from a normal distribution and having six repeated measures. We fixed the effect size of the virtual covariate to be medium (Cohen’s f2 = 0.15), and varied the effect sizes of the sleep pattern variable and the GM diversity variable between medium (f2 = 0.15) and large (f2 = 0.35) as were observed in unpublished preliminary data. Figure 2 shows that 160 infants are required to achieve a power of 0.8 to detect a significant medium-sized effect at α = 0.05. Adjustment for a 20% attrition rate requires that we recruit 192 mother-infant pairs.

### Participants and procedures

#### Maternal sample subject eligibility

Inclusion criteria are (1) English- and/or Spanish-speaking mothers in their 3rd trimester of pregnancy through 3 weeks postpartum from the Phoenix, Arizona community; (2) ages 18 to 40 y; (3) with a telephone contact; and (4) not intending to move from the area for at least 1.25 years. Exclusion criteria are (1) mothers who have chronic metabolic diseases (e.g., types 1 and 2 diabetes prior to pregnancy, cardiovascular disease) that impact fetal growth; (2) experienced a high-risk pregnancy (e.g., preeclampsia, HIV infection); (3) delivered a small or large newborn (< 2500 g or > 4000 g); (4) hospitalization after discharge of infant; (5) separation from infant; (6) experiencing significant postpartum complications; (7) report alcohol, tobacco, and marijuana use in the second and/or third trimesters [53]; or (8) report illicit substance use during any trimester.

#### Infant sample subject eligibility

Inclusion criteria include full-term (≥37 weeks of gestation) singleton infants who have no growth abnormality or other endocrine, chromosomal, or genetic abnormality, or severe illness-related comorbidity that could impact growth and development. Exclusion criteria are (1) mother-infant pairs who are not discharged together from the hospital; or (2) infants born with congenital abnormalities or conditions that may be expected to result in developmental delays or birth weights < 2500 g or > 4000 g.

Potential participants will be identified by providers and research staff through in-house recruitment from obstetrics and pediatric units at a local public hospital system, and a network of Supplemental Nutrition Program for Women, Infants, and Children (WIC) clinics. Advertisements will also be distributed through social media, Arizona State University (ASU) Institutional listservs, word-of-mouth, sharing flyers with active study participants, community events, and health professional referrals from clinical and community partners. Eligible participants will provide their contact information. Regular telephone calls, emails, or text messaging contacts will be made to determine birthdate, if eligibility is maintained, and to schedule the first in-home visit. Secondary (e.g., family member, friend) contacts will be obtained in case participants lose phones or change numbers. Enrollment and consent will occur at the first in-home visit at 3-weeks post-delivery.

**Fig. 3:**
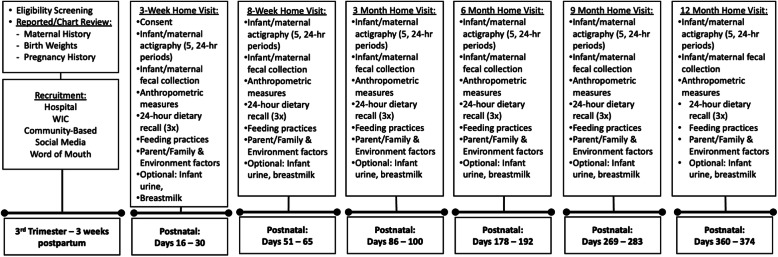
Flow chart of study procedures

Study procedures are designed to capture periods during which important neurodevelopmental milestones tend to occur, which have also been associated with early life adiposity [53] (Fig. 3). In-home visits will be conducted in near time to 3 and 8 weeks, and 3, 6, 9, and 12 months postpartum. Along with major neurodevelopmental milestones that tend to occur at these ages, important shifts in infant feeding [54–56], sleep-wake pattern development [57–61], and GM community changes that have previously been associated with infant and childhood weight status [34, 37, 62–64] tend to coincide with these time periods.

At all home visits, research staff who speak English and Spanish will assess infant anthropometrics, administer brief sleep and health-related questionnaires and explain how to complete a 3-day diet record, instruct the mother on proper retrieval of infant and maternal fecal, infant urine, and maternal breast milk samples, and provide mothers and infants with wrist and ankle actigraphs, respectively, to capture sleep data. After data collection, participants will mail back actigraphs, sleep diaries, and dietary recall forms in pre-stamped boxes. The first participant was enrolled on 09 Nov 2020. Recruitment and follow-up assessments are ongoing.

Procedures were adapted to account for the COVID-19 pandemic such that data collection could be conducted through primarily electronic delivery (e.g., video conferencing, instructional videos, online or telephone survey administration), drop-off and curbside pick-up of research equipment and samples, and/or outdoor or social distanced interactions at participant homes. On the day of the home visit, research staff will take their temperature and complete a COVID-19 symptom self-attestation survey maintained by the study investigators. Participants will also complete a COVID-19 symptom attestation survey for themselves, their infant, and all members of their household prior to the visit. Staff will maintain appropriate social distancing measures while interacting with participants, and use personal protective equipment including masks, gloves, and hand sanitizer. If the mother would prefer for study staff to not have physical contact with the infant, the staff will instruct the mother how to accurately measure their baby under the staff’s supervision. After the visit, the staff will disinfect surfaces that have come into contact with participants (e.g., weight scale) before packing up all study equipment.

### Assessment of RWG and infant growth

RWG is the primary outcome and weight gain over 12 months is a secondary outcome in this study. Infant length and weight will be measured with portable recumbent length boards (nearest mm) and electronic digital scales (nearest 0.1 kg), respectively. Infants will be nude at the time of weighing and will be measured recumbent to maintain measurement consistency. Weight measures will be recorded on the weight-for-age (W/A) percentile growth grid from the National Center for Health Statistics [65] to determine body mass in children under 2 years of age. W/A percentiles will be converted to Z-scores to allow group comparison by sex. Infant head circumference will be measured to the nearest mm using a flexible tape measure (Hopkins Medical Products) designed for infant heads. Infant waist circumference will be measured to the nearest mm using a Gulick II tape measure with tension spring.

### Assessment of sleep

Infant and maternal sleep will be measured with retrospective questionnaires and prospective actigraphy and sleep diaries. The extended Brief Infant Sleep Questionnaire (BISQ; 24 items) will evaluate infant bedtime problems, excessive sleepiness, awakenings, and sleep practices (e.g., bed/room-sharing, sleep position) [66, 67]. This questionnaire has been validated against sleep diaries and actigraphy [67]. To assess the mother’s sleep we will use the Pittsburgh Sleep Quality Index (PSQI), a 19-item questionnaire that measures sleep quality over the past month [68]. It has demonstrated reliability and construct validity among pregnant and postpartum mothers [69–71].

Actigraphy is a standard, valid, noninvasive measure for assessing sleep and circadian rhythm patterns across the life course [72]. We will use the Micro Motionlogger actigraph (Ambulatory Monitoring Inc., Ardsley, New York) that will be analyzed using validated sleep-wake scoring algorithms for infant sleep [73] and adult sleep [74]. The infants and mothers will wear the devices for 5 continuous days and nights after each study visit [75, 76] to capture the full development of sleep-wake patterns and circadian rhythmicity across infancy and the level of synchrony with maternal sleep-wake patterns. Primary sleep variables will be averaged across the recording period and include (1) total sleep minutes, excluding awakenings from sleep onset to final sleep bout awakening per 24 h, (2) sleep efficiency or percent of actual sleep minutes divided by the time interval from sleep onset to morning awakening per 24 h, (3) the number of long awakenings (≥5 min) during the nocturnal sleep period, (4) longest continuous sleep interval, and (5) nocturnal sleep onset time after parent-reported bedtime. Circadian rest-activity rhythmicity will be derived from cosinor analysis by which a cosine curve is applied to the data with linear regression over a specified 24 h, as well as non-parametric circadian rhythm analysis (NPCRA) to account for the presence of ultradian rhythms and to improve estimates of sleep-wake rhythmicity in newborns [77, 78]. Primary sleep-wake circadian rhythm variables from cosinor analysis with a fixed 24-h cycle will be mesor (mean activity count), magnitude (difference between mesor and peak amount of activity), acrophase (clock time of peak activity), and R^2^ (variance explained by the cosine fit or how well the data fit a 24-h circadian rhythm), and primary variables from NPCRA will be L5 (clock time of lowest activity), M10 (clock time of highest activity), amplitude (difference between L5 and M10), inter-daily stability (measure of similarity between days of rest-activity pattern; values range from 0 to 1.0 with greater values indicating greater stability), and inter-daily variability (measure of fragmentation of the rest-activity patterns) [78].

Mothers will also complete sleep diaries on the sleep of their infants and their own sleep. Diaries will be used to identify artifacts that might affect motion (e.g., swings, car seat, swaddling) and to define sleep periods from actigraphy output for infants and mothers prior to analysis with the sleep-wake algorithms [79]. Primary sleep diary variables will be (1) bed/nap clock times attempted to sleep, (2) bed/nap clock times of sleep bout awakenings, and (3) time in bed or interval between attempted bed and wake times.

### Fecal sample collection

Mothers and infants will be asked to collect fecal samples at each study timepoint using sterile swabs (HydraFlock Double Flocked Swab #25–3306-2H BT, Puritan, Guilford, ME). Maternal fecal samples will be collected by swabbing soiled toilet paper. Infant samples will be collected from soiled diapers. Date and time of collections will be recorded by the mothers. Supplies will be provided to mothers the week before planned in-home visits with instructions to collect that day’s sample from the infant’s diaper and immediately store it on ice in an insulated cooler. Study staff will pick up tubes within 24 h of collection and transport them on ice to the laboratory for storage at − 80 °C until processing.

Soiled infant diapers will be collected for additional secondary analyses including metabolomics and exfoliomics analyses (see below for details). Diapers will be kept on ice in an insulated cooler and transported to the laboratory for processing.

### Assessment of gut microbiome

Microbial genomic DNA will be extracted from fecal samples using DNeasy PowerSoil Pro DNA isolation kits and a vortex-based beadbeater, the recommended method of the NIH Human Microbiome Project and previous used by our team [80]. The hypervariable V4 region of the 16S rRNA gene will be amplified from stool samples using barcoded 806R and 515F primers [81] and 5 Prime Hot MasterMix (5 Prime, Germany) in triplicate. Quality of the amplicons and potential contamination will be checked on an agarose gel. Amplicons will be quantified using Picogreen (Invitrogen), according to the manufacturer’s protocol. A total of 200 ng of amplified DNA from each sample will be pooled and cleaned using UltraClean PCR Clean-Up Kit, and then diluted, denatured (0.2 N NaOH), and sequenced on the MiSeq platform (Illumina), as previously described [81]. Due to the limited sequence diversity among 16S rRNA gene amplicons, 10% of the PhiX control library (Illumina) made from phiX174 will be added to the run. A 7 pM aliquot of the pooled 16S rRNA gene library will be subjected to paired-end sequencing using 2 × 250 bp MiSeq Reagent Kit V2 (Illumina). Sequencing will be performed by the Genomics Core at ASU using the Illumina MiSeq and MiSeq Control Software. Pooled sequences will be de-multiplexed and quality filtered using the QIIME2 software package [82]. Sequences will be assigned to operational taxonomic units (OTU) with a 99% similarity threshold using QIIME’s uclust-based open-reference OTU picking protocol against most recent SILVA or GreenGenes reference databases. Sequences that do not match the reference database will be clustered de novo; thus, all sequences will be included in the analysis. Core diversity analyses will be performed on the OTU tables, including alpha and beta diversity as well as taxonomic summaries as provided by QIIME software.

### Intestinal exfoliated cell transcriptome assessment

Stool specimens from infant diapers will be obtained using sterile tongue depressors to allow for assessment of transcript expression in sloughed host epithelial cells. Approximately 10 g of fresh stool will be transferred to a sterile 50 ml conical tube containing 10–20 ml of DNA/RNA Shield reagent (Zymo Research Corp, Irvine, California, USA) to stabilize nucleic acids. Prior to immediate storage at − 80 °C, samples will be mixed to a homogeneous slurry by hand using a sterile conical tube pestle. Samples will be shipped on dry ice to Texas A & M University for subsequent processing and analyses as previously described [83–85].

### Fecal metabolomics analysis

Approximately 120–600 mg of stool collected from soiled infant diapers will be aliquoted and flash-frozen at − 80 °C in sterile 2 mL microcentrifuge tubes until further analysis, as previously described [86]. For fatty acid extraction, fecal metabolite samples will be extracted with chloroform:methanol (2:1, v/v) after adding an internal standard (myristic acid-d27). For aqueous extraction, metabolites will be extracted using a mixture of 80:20 (v/v) methanol:water that contains methyl succinate as the internal standard. Aqueous global profiling experiments will be performed using Agilent 7820 GC-5977 MS and Thermo Scientific Electron Orbitrap Elite Velos Pro UPLC-MS instruments. To identify peaks from the MS spectra, we will make extensive use of the robust NIST and Fiehn’s libraries for GC-MS, and HMDB metabolite library and METLIN database for LC-MS experiments. We will follow general procedures for Agilent Fiehn GC-MS Metabolomics RTL library [87, 88], with minor changes incorporated to improve detection sensitivity.

Short chain fatty acids (SCFA) will be assessed using standard MS operating procedures [89]. In brief, infant fecal samples (50 mg) will be spiked with internal standard (caproic acid-6,6,6-d3, 200 μM in H_2_O, 20 μL), and then homogenized in 20 μL sodium hydroxide solution (NaOH, 0.5 M in water) and 480 μL methanol (MeOH). Afterwards, another 400 μL MeOH will be added (pH ~ 10), and upon storage at − 20 °C for 20 min, 800 μL of supernatant will be collected. Samples will be then evaporated to dryness, reconstituted in 40 μL of methoxyamine hydrochloride in pyridine (20 mg/mL), and stored at 60 °C for 90 min. Afterward, 60 μL of N-Methyl-N-tert-butyldimethylsilyltrifluoroacetamide will be added and stored at 60 °C for 30 min. SCFAs will be detected on an Agilent 7820A GC 5977 MS system installed with a HP-5 ms fused-silica capillary column (30 m × 0.25 mm × 0.25 μm; Agilent J&W Scientific, Folsom, CA).

Targeted LC-MS/MS measurements of infant fecal bile acids will be completed [90, 91]. Fecal samples (50 mg) will be spiked with internal standards (ISs, 10 μM of LCA-D4, DCA-D4, CA-D4, GCDCA-D4, and GCA-D4), then homogenized, protein precipitated, dried, and reconstituted in 100 μL methanol/water (50:50, v/v). To assess 56 bile acids, 2 μL of each sample will be injected into an Agilent 1290 UPLC-6490 MS/MS system using negative ionization mode and a Waters XSelect HSS T3 column (2.5 μm, 2.1 × 150 mm) for chromatographic separation. To determine absolute bile acid concentrations, calibration curves will be constructed for bile acid standards in reference to corresponding ISs and concentrations calculated from peak areas and calibration curves.

### Breastmilk sample collection

Human milk samples will be collected from breastfeeding mothers at up to six of the study timepoints, depending on when breastfeeding is stopped during the first year of each infant’s life. Women will be asked to hand express or pump approximately 15 mL of milk into a 50 mL sterile conical tube and store it on ice in an insulated cooler. Within 24 h of collection, samples will be transported by study staff to the laboratory for long-term storage at − 80 °C until further processing.

### Milk microbiome assessment

To assess the microbial composition of human milk samples, microbial DNA will be extracted from samples using the Quick-DNA Fungal/Bacterial Miniprep Kit (Zymo Research Corp, Irvine, California, USA), after the milk fat is removed using an adapted centrifugation protocol and disposable inoculation loop [92]. The extraction process will follow the manufacturer-recommended protocol from Zymo Research Corp. with adjustments as previously specified [92]. Milk microbiome sequencing will be performed as written for fecal samples above, after amplifying microbial DNA using V4 primers for the 16S rRNA gene.

### Infant urine collection

A minimum of 5 mL of infant urine will be collected at each of the six study visits using BPA-free pediatric urine specimen collection bags (U-Bag, Hollister, Inc., Libertyville, Illinois, USA). Collected urine will then be transferred to 30 mL Nalgene containers and stored on ice in an insulated cooler. Within 24 h of collection, study staff will transport the samples on ice to the laboratory where they will be stored at − 80 °C.

### Endocrine disruptor exposure analysis

Urine samples will be thawed and endocrine-disrupting chemical (bisphenols, phthalates, etc.) concentrations will be assessed using high performance liquid chromatography with tandem mass spectrometry as previously described [93]. Specific gravity will be measured in all urine samples to account for differences in analyte dilution.

### Assessment of covariates

Covariates from each EMG construct (Fig. 1) will include child characteristics, maternal/family characteristics and context, feeding practices and dietary intake of infants and mothers, and home and care environmental factors/exposures. Interactions among EMG constructs also will be assessed, including maternal-child interactions and feeding interactions. An overview of the measures and their relation to ecological constructs are provided in Table 1.

**Table 1:** Summary of study variables and measures, by Ecological Model of Growth construct

			Prepartum	Postpartum					
Ecological Construct	**Variable/Type**	**Measures/Instruments**	**Pregnancy (3rd trimester)**	**3 wk.**	**8 wk.**	**3 mo.**	**6 mo.**	**9 mo.**	**12 Mo.**
Child (Host/Agent)	Birth History	Gestational age, method of delivery, birth weight & length, day 2–5 weight & length, duration of labor, antibiotic exposure (from medical chart & reported)		✓					
	Child demographics	Sex, race/ethnicity		✓					
	Medical History	Antibiotic use, illnesses, other medications		✓	✓	✓	✓	✓	✓
	Infant Temperament	Infant Behavior Questionnaire – very short form					✓		
	Reported Sleep	Brief Infant Sleep Questionnaire and parent-reported sleep diaries (5 days each		✓	✓	✓	✓	✓	✓
	Objective Sleep	Ankle actigraphy (5 days each)		✓	✓	✓	✓	✓	✓
	Food Allergy	FDA and CDC Infant Feeding Practices study II						✓	
	Gut microbiome and metabolomics	Soiled diaper & swabs (Microbial genomic DNA and metabolites)		✓	✓	✓	✓	✓	✓
	Intestinal cell transcriptomics (exfoliomics)	Soiled diaper (Microbial genomic RNA and human intestinal cell RNA)		✓	✓	✓	✓	✓	✓
	Exposure to nutrients, environmental phenols, and phthalates *	Urine sample with urine collection bag		✓	✓	✓	✓	✓	✓
	Anthropometrics	Weight and length on standardized instruments		✓	✓	✓	✓	✓	✓
Maternal & Family	Maternal demographics	Age, education, race/ethnicity, income, nativity, length of time in U.S., occupational status, shift work status, parity, birth spacing,	✓	✓					
	Maternal pregnancy factors	Pre-pregnancy body mass index, gestational weight gain, complications, medication, antibiotic exposure, alcohol/tobacco exposure (from medical chart & reported)	✓	✓					
	Acculturation	Brief Acculturation Rating Scale for Mexican Americans		✓					
	Employment changes	Return to work timing, shift work status			✓	✓	✓	✓	✓
	Discrimination	Everyday Discrimination Scale			✓				
	Maternal distress	Edinburgh Postnatal Depression Scale; Depression Anxiety Stress Scales-21		✓	✓	✓	✓	✓	✓
	Maternal knowledge/intentions for infant feeding	Breastfeeding Attrition Prediction Tool	✓						
	Maternal sleep	Wrist actigraphy, sleep diaries, Pittsburgh Sleep Quality Index		✓	✓	✓	✓	✓	✓
	Maternal beliefs about infant sleep	Maternal Cognitions about Infant Sleep Questionnaire				✓		✓	
	Gut microbiome *	Double-tip swabs (Microbial genomic DNA)		✓	✓	✓	✓	✓	✓
	Breastmilk microbiome *	Manual or breast pump expression		✓	✓	✓	✓	✓	✓
Feeding Practices & Dietary Intake	Feeding Practices (infants)	Breastfeeding duration, exclusivity, intensity mixed feeding, formula type, etc. via FDA and CDC Infant Feeding Practices study II		✓	✓	✓	✓	✓	✓
	Dietary Intake (mothers and infants)	Introduction to solid foods and early infant feeding amount/duration via direct query & 3 self-reported dietary records (evaluated with Minnesota Nutrition Data System for Research (NDSR))		✓	✓	✓	✓	✓	✓
Environment	Home environment	Home Observation for the Measurement of the Environment Inventory (HOME)			✓		✓		✓
	Daycare environment	FDA and CDC Daycare Arrangements and Feeding Practices			✓	✓	✓	✓	✓
	Food security	USDA Household Food Security Survey		✓			✓		✓
	Tobacco exposure	Direct query			✓				
	Infant sleep environment	Brief Infant Sleep Questionnaire (e.g., bed-sharing, sleep position)		✓	✓	✓	✓	✓	✓
Maternal-Child Interactions	Maternal-child interactions	Home Observation for the Measurement of the Environment Inventory (HOME), Postpartum Bonding Questionnaire			✓		✓		✓
	Bedtime interactions	Parental Interactive Bedtime Behaviors Scale				✓		✓	
Feeding Interactions	Maternal-child feeding interactions	Infant Feeding Style Questionnaire					✓		✓

*CDC* centers for diseases control and prevention, *FDA* food and drug administration

* Measure will be collected at each visit if the participant opts into these biospecimen collections

Other child characteristics beyond infant sleep, gut microbiome/exfoliome, and anthropometrics that will be assessed will include child demographics, birth history and anthropometrics (mother-reported), medical history throughout the study period (including antibiotic exposure), infant temperament with the Infant Behavior Questionnaire - Very Short Form [94], food allergies (adapted from the Food and Drug Administration and Centers for Diseases Control and Prevention Infant Feeding Practices Study II (IFPS II)) [95], and levels of endocrine disrupters via a urine sample.

Maternal characteristics with clear associations to infant RWG or GM development will be assessed. These include pre-pregnancy BMI, gestational weight gain, parity, birth spacing, pregnancy history (e.g., route of delivery, antibiotics for group B Streptococcus), pregnancy complications, medical history and health throughout the study (including antibiotic exposure), acculturation via the Brief Acculturation Rating Scale for Mexican Americans [96] which also has been used in other Spanish speaking populations, depression via the Edinburgh Postnatal Depression Scale [97, 98], stress and anxiety via Depression Anxiety Stress Scales-21 [97, 99], infant feeding knowledge and intentions via the Breastfeeding Attrition Prediction Tool [100, 101]. Other maternal factors that remain unclear with regard to RWG will also be explored in analyses, including age, race/ethnicity, education, nativity, length of time in the U.S., employment status, shift work status, return to work timing [102], perceived discrimination via the Everyday Discrimination Scale [103], perceived parental competency via the Parental Sense of Competence Scale [104], Maternal Cognitions about Infant Sleep Questionnaire [105], and COVID-19 pandemic experiences during pregnancy and immediately postpartum with the Environmental Influences on Child Health Outcomes (ECHO) Impacts of the COVID-19 Outbreak on Pregnancy - Recall questionnaire [106].

Family characteristics and contextual factors will be assessed for associations with RWG. Included factors will be number of other household members (including children), sibling order for infant, household socioeconomic status via income and occupation, and father presence.

Feeding practices and dietary intake of infants and mothers will be evaluated via three self-reported dietary records (one weekend day and two weekdays). Mothers will complete dietary records around the time of each of the six study visits. They will be asked to report on the type, frequency and quantity of foods, beverages and supplements consumed on each day. Household comparisons will be provided with each food record to assist with estimating food portion sizes. Staff will review each record and probe for missing items (e.g., beverages, condiments, etc.). During the study visit when mothers first report solid food consumption for their infants, mothers will also be asked to complete three diet records for their infants. This will be done for two weekdays and one weekend day and will be used to define the type, quantity, and frequency of foods, beverages, and supplements being consumed by participating infants. Diet records for each mother and infant will be completed for all remaining study visits. The University of Minnesota Nutrition Data System for Research (NDSR) will be used to evaluate the nutritional composition of foods and beverages consumed by mothers and infants. Mothers will also be asked to report on breastfeeding and infant feeding practices using questions adapted from the validated questionnaires of the Food and Drug Administration and Centers for Disease Control and Prevention IFPS II [95]. In brief, mothers will report on infant dietary intake prior to solid food introduction, including breastfeeding duration and the quantity and brand of formula and/or solid foods consumed over a 24-h period. Changes in feeding practices since the last visit will be assessed including formula products containing prebiotics or probiotics to account for differential effects on the GM compared to standard formulas; solid food introduction timing, type, and amount; mode of consumption (e.g., breast, bottle, cup); and scheduled vs. on-demand feeding. Breastfeeding intensity will also be assessed at each visit [107].

Home and care environment factors that will be measured will include the Home Observation for Measurement of the Environment (HOME) Inventory [108], parent-reported daycare environment (if present) including feeding and sleep environment arrangements within the care environment, with the Daycare Arrangements and Feeding Practices [95], and familial food insecurity with the USDA Household Food Security Survey [109], household tobacco exposure [110], and infant sleep environment via the Brief Infant Sleep Questionnaire [67],

Maternal-child interactions also will be measured with the Home Observation for Measurement of the Environment (HOME) Inventory [108], as well as with the Postpartum Bonding Questionnaire [111], and the Parental Interactive Bedtime Behaviors Scale [112]. Feeding interactions will be measured with the Infant Feeding Style Questionnaire [113].

### Ethical considerations

The study protocol was approved by the Valleywise Health Institutional Review Board (2019–060). On the consent, participants will be given as much time as needed to understand the purpose of study, required procedures, benefits and risks of their participation, and ask questions. Voluntary participation and anonymity will be emphasized. Participants will sign and date consent. They will also be asked to opt-in and sign a Social Media Etiquette Agreement for appropriate use of study social media accounts to interact as a community of mothers. Participants will be provided payment at the completion of study assessments associated with each home visit and phone calls between visits for the purposes of monthly check-ins and 24-h dietary recall. The total amount of money that can be earned for completing study-related tasks is $234. In addition to compensation, at each visit mothers and infants will also be given a “swag bag” of items of particular relevance to this population (e.g., diapers, wipes, custom-made baby milestone books, teethers, etc.). Additional incentives will be offered to participants which will include quarterly raffle prizes (small denomination gift cards and study-branded items) and incentives for friend referral ($5/friend for up to 5 friends; $25 total).

### Statistical analyses

To evaluate Aim 1, visit-to-visit changes in 24 h sleep and sleep-wake rhythmicity variables and their relationship to occurrence of RWG will be examined with a logistic TVEM. It can reveal critical periods during which changes in sleep-wake patterns accelerate/decelerate infant weight gain [114] and include other covariates with time-invariant effects. The TVEM model can be formulated as,

1 $$ logit\;(RWG)={\beta}_0f(t)+{\Sigma}_v\kern0.5em {\beta}_vf(t)V+{\Sigma}_c\kern0.5em {\beta}_cC+\in $$

where *f*(*t*) is a smooth function of time *t*, *V* is a set of variables with time-varying effects, *C* is a set of variables with time-invariant effects, *β*_0_, *β*_*v*_ and *β*_*c*_ are the corresponding coefficients, and *ϵ* is normally distributed random errors. We will impose no constraints on the *f*(*t*) function and the *β* coefficients except an assumption of smooth temporal progressions. To fit the model given the collected data, we will use the %TVEM SAS macro [115] and use a P-spline method to select the best-fitting model using knots (splitting points). Significance will be assessed based on the *p*-values of estimated *β* coefficients.

To evaluate Aim 2, we will apply the TVEM in model (1) with representing the alpha and beta diversity scores for each taxon. Because there are hundreds of taxa that may share dependencies, we will compute false discovery rates (FDRs) using the Benjamini–Hochberg procedure [116]. Taxa with FDR < 5% will be regarded as having significant associations. Given the hierarchical structure among taxa, we will further reduce the redundancies among significant taxa by ranking them on Akaike information criterion (AIC) values. Specifically, we will traverse the taxonomic tree (leaves to root) and compute AIC values for each clade. If a parent node has a lower AIC value than any of its child nodes, we will keep the parent node and remove the child nodes from the short-listed taxa.

To evaluate Aim 3, exploratory analysis will apply principal component analysis to examine if the combination of GM variables and sleep-wake variables can explain a higher variance of RWG than using each class of variables independently. Confirmatory analysis will use model (1) to include the main effects and pairwise interactions between GM diversity scores and sleep-wake variables. Significant p-values and positive coefficients of interaction terms will indicate synergistic effects. However, because there are potentially many GM taxa associated with RWG, model (1) may include too many variables and trivial effects that reduce the statistical power. In this case, we will take a nested approach to test synergies among correlated variables. Specifically, combining the significant variables identified in Aims 1 and 2, we will first test pairwise correlations. We will then group significantly correlated variables using Gaussian finite mixture models [117] that automatically determine the number of clusters. If GM diversity scores and sleep-wake patterns coexist in a cluster, we will use model (1) extended with interaction terms to test synergistic effects. For the proposed exfoliomic data, we will utilize systems biology approaches to illuminate transgenomic cross-talk between host exfoliated intestinal epithelial cells [84] and the genomes of the gut microbiota [83, 118] in a longitudinal cohort.

## Discussion

Excess weight for infant age and childhood obesity remain highly prevalent with significant potential for negatively impacting growth, development, and health outcomes across the life course. Early infant RWG is a meaningful, early heralding risk factor for subsequent adiposity [119–121]. While prenatal exposures and feeding-related predictors of early infant RWG are known [122–125], they do not wholly explain the variance in accelerated weight gain. Less is known about other modifiable factors in early life that may be protective or detrimental. Suboptimal 24-h sleep-wake and commensal GM development and maturation are associated with subsequent adiposity in childhood and adulthood, yet their relationships with infant RWG are less known. In our Snuggle Bug/Acurrucadito Study, we aim to investigate sleep-wake and GM development across infancy in association with subsequent RWG independently, examine the temporal crosstalk between sleep-wake pattern and GM development, and explore whether these interactions affect RWG propensity. There are several noteworthy aspects in this protocol. The Snuggle Bug/Acurrucadito study will identify multidimensional factors that may contribute to the development of childhood obesity, especially among high-risk populations such as those with greater maternal BMI and racially and ethnically-diverse mothers of low-income. This study proposes a novel EMG theoretical framework to ensure a comprehensive understanding about the interacting socio-environmental, sleep, and infant GM influences on infant adiposity outcomes. We are employing a prospective longitudinal approach to assess multiple objective and self-reported parameters at clinically meaningful intervals throughout the first 12 months of life that will provide robust and accurate measures of sleep, GM, growth, and the maternal-infant, familial, and environmental context. The non-invasive longitudinal fecal analyses will allow us, for the first time, to identify critical molecular biomarkers that define the relationship between establishment of 24-h sleep-wake patterns, weight gain trajectories, early nutrition, the intestinal microbiota and intestinal gene expression. Our TVEM with frequent sampling approach in early infancy will capture the functional form of growth and aid in elucidating the dynamic associations between study variables and RWG. Further, to reduce language barriers and enhance our ability to recruit our target high risk population, all study measures to be collected will be translated by bicultural and bilingual staff, in either English or Spanish. All staff conducting home visits are able to communicate in English and Spanish. With these unique strengths, the study will provide a wealth of findings that will advance population and clinical-level approaches to childhood obesity prevention.

Some potential challenges should be acknowledged. Recruitment and retention of participants in longitudinal research is often challenging. To address this challenge, at recruitment and each in-home visit, we will offer “swag” bags with items of interest to pregnant and postpartum mothers and their infants. Regular contact with a designated staff member will establish strong relationships to help aid retention over the study period of 12–15 months*.* We will further incentivize regular contact for each answered call, every time a mother notifies the team of a change in contact information, and for each 24-h dietary recall completed by phone. Mothers will be reminded by telephone or postcard of their next scheduled home visit. We will also send participants birthday, major holiday, and Mother’s Day cards. Further, mothers will be given the opportunity to opt-in to a study-developed, social media-based community of other participants to share in their experiences as mothers of young children. Additionally, sampling bias might occur if individuals worry about the risks of in-home visit due to the COVID-19 pandemic and shy away from participation. In order to minimize the risk of sampling bias and maximize the validity of the study, recruitment efforts will emphasize the numerous safety precautions and social distancing measures that the study can employ to minimize any in-person interactions. Lastly, mothers might hesitate to collect fecal and other biobehavioral measures. The research team has prepared and uploaded written and video instructions to the study website, including the guidelines to collect all biospecimens at home for mothers and infants. Additionally, nitrile gloves will be provided for mothers to use when collecting biological specimens, and proper hand washing instructions will be provided to minimize the risk of fecal bacterial exposure.

Through the identification of optimal and crucial developmental intervals for targeting and preventing RWG, this observational, longitudinal study will provide evidence to spur the development of theoretical and data-driven interventions to prevent RWG and subsequent obesity, and clinical practice guidelines that address modifiable factors that influence sleep-wake and GM development in support of optimal metabolic and growth outcomes.

## Data Availability

Data sharing is not applicable to this article as no datasets were generated or analyzed given this described a protocol for a study.

## Abbreviations

AIC: Akaike information criterion
ASU: Arizona State University
BISQ: Brief infant sleep questionnaire
BMI: Body mass index
EMG: Ecological model of growth
FDRs: False discovery rates
GM: Gut microbiota
HOME: Home Observation for Measurement of the Environment (HOME) Inventory
NDSR: Minnesota nutrition data system for research
NPCRA: Non-parametric circadian rhythm analysis
OUT: Operational taxonomic units
PSQI: Pittsburgh sleep quality index
RWG: Rapid weight gain
TVEM: Time-varying effect model
W/A: Weight-for-age
WIC: Women, infants, and children
